# Does Hamstring Graft Size Affect Functional Outcome and Incidence of Revision Surgery After Primary Anterior Cruciate Ligament (ACL) Reconstruction?

**DOI:** 10.7759/cureus.21158

**Published:** 2022-01-12

**Authors:** Nuthan Jagadeesh, Tushar Dhawan, Fahim Sheik, Vishwanath Shivalingappa

**Affiliations:** 1 Trauma and Orthopaedics, Vydehi Institute of Medical Sciences and Research Centre, Bangalore, IND; 2 Orthopaedics, Vydehi Institute of Medical Sciences and Research Centre, Bangalore, IND; 3 Orthopaedic Surgery, Vydehi Institute of Medical Sciences and Research Centre, Bangalore, IND

**Keywords:** size of graft, functional outcome of acl reconstruction, knee injury and osteoarthritis outcome score, acl tear, anterior cruciate ligament reconstruction, hamstring graft

## Abstract

Purpose

The primary outcome measure of this study was to determine the effect of hamstring graft size on the functional outcome of arthroscopic anterior cruciate ligament reconstruction (ACL-R) and the secondary outcome was to ascertain the effect on revision surgery at the two-year follow-up.

Methods

This is a prospective comparative study of 144 consecutive patients undergoing primary ACL reconstruction using a hamstring autograft. All patients underwent graft harvesting and ACL reconstruction with the standard technique. The graft diameter was recorded intraoperatively using a graft sizer. All patients were followed up with the Knee Injury and Osteoarthritis Outcome Score (KOOS) recorded at preop, six weeks, six months, one year, and two years and whether they underwent revision during this period was documented.

Results

The mean KOOS for patients with a ≤ 7mm graft diameter was 80.5±13.1, which was significantly lower compared to those with graft > 7 mm of 88.3±8.5, respectively (p<0.001) at the two years follow-up. Patients with graft ≤ 7mm did poorly, especially with mean KOOS subscores of sports and recreation and quality of life (p<0.05). Twenty-three point one percent (23.1%; 3 out 13) of patients with a graft < 7mm underwent revision, whereas only 5.8% and 2.6% of patients underwent revision with a graft diameter of 7.1-8.0 and 8.1-9.0 (p=0.027).

Conclusions

The smaller Hamstring graft diameter leads to poorer functional outcomes of the patient’s ACL reconstruction. Though the number of revisions was high among those with a graft diameter of ≤ 7mm, multicentric studies with many revisions are required to confirm the relation.

## Introduction

The incidence of anterior cruciate ligament (ACL) injuries has increased, and its surgical reconstruction has become a routinely practiced procedure worldwide by many orthopedic surgeons over the past two decades [1-2]. The absolute aim of the surgeon performing an ACL reconstruction is to achieve normal functionality of the knee, and ensure that the patient resumes a routine lifestyle and returns to sports and recreation [3-6]. Even with a well-documented success rate, anterior cruciate ligament reconstruction (ACL-R) surgery has witnessed pitfalls, as many patients undergoing the procedure must invest a significant amount of time and effort for rehabilitation especially in those involved in sports activities [7-8].

Various graft options exist for ACL reconstruction like hamstring, bone-patellar tendon, quadriceps, etc., and multiple factors influence finalizing the decision of the ideal graft choice for the patient. It depends on whether the graft can re-create the anatomical and biomechanical properties of the native ligament, assures safer fixation, faster biological integration, and minimal donor site morbidity. Also, the training and experience of the surgeon harvesting a particular graft are important [4,9-10]. Hamstring grafts involving semitendinosus and gracilis tendons (STG) are among the most used grafts for ACL-R. The advantages of hamstring graft are that it is easy to harvest and has good strength with lower donor site morbidity. However, the major limiting factor associated with the hamstring tendon is the inability to predetermine a mean score size of the graft before surgery, unlike that seen with the bone-patellar tendon, quadriceps tendon, and allografts, which can be predetermined [11-12]. Several studies have tried determining the relationship of the size of hamstring grafts with patient parameters, such as height, weight, age, sex, and body mass index (BMI), and utilized this information to predict graft size preoperatively [13-14]. Moreover, the cross-sectional area of STG tendons on magnetic resonance imaging and ultrasound has also been used to predict HS graft diameter [15-16], but the reliability and consistency of these findings to accurately predict size has been questionable.

Revision of ACL reconstruction is technically demanding with poor outcomes and its incidence is found to decrease with an increase in the graft size. Graft size is considered one of the major determining factors in predicting the revision rates in ACL-R procedures [17-19]. Graft size less than 8 mm in a young patient less than 20 years is said to be commonly associated with revision surgeries. Graft size ≥ 8 mm is said to be associated with a better outcome of the ACL-R surgery and prevents the need for a future revision of ACL-R [20-22]. However, a limited number of studies determine the influence of graft size on the functional outcome of the patient. There could be many people with poor functional outcomes but not undergoing revision surgeries owing to the lesser success rate of revision surgeries. Hence, considering revision surgeries alone may not determine the true influence of the size of graft on the success of ACL reconstruction.

Hence, the primary outcome measure of this study was to determine whether the graft size of the hamstring graft (STG) plays a role in determining the functional outcome of arthroscopic anterior cruciate ligament reconstruction and the secondary outcome measure is to determine whether it increases the need for revision surgery at the two-year follow-up.

## Materials and methods

Among 233 patients, 144 who underwent isolated ACL reconstruction using a hamstring autograft between January 2015 to January 2020 in our hospital were included for evaluation. Patients with a multi-ligament injury, associated meniscal tear requiring intervention, previous trauma to the knee, bilateral ACL tear, revision surgeries, neuromuscular disorder, associated neurovascular injury, and graft amputation while harvesting the graft were excluded. Patients who were not available for follow-up for at least one year were also excluded. All patients included underwent ACL reconstruction using a hamstring graft with either a bio screw/titanium on the tibial side and an Endobutton on the femoral side as a fixation device. An accessory anteromedial portal with a femoral and tibial jig was used for the tunnel. All patients underwent surgery with valid informed consent. Institutional ethical committee approval was taken to conduct the study. Demographic data and clinical findings were documented, and MRI was used to confirm the diagnosis of all patients. Preoperative functional knee scores were documented using the Knee Injury and Osteoarthritis Outcome Score (KOOS) scoring system.

Operative technique

All grafts were harvested by the senior surgeon or in his presence by other fellowship-trained specialists with more than two years of experience of doing ACL reconstruction using a 2-3 cm vertical anteromedial incision on the proximal tibia, at the level of the insertion of the semitendinosus and gracilis muscles. Following this, the tendon of the STG muscle was dissected individually, stripped against the bone, and the ends were whip-stitched using a non-absorbable 2-0 Ethibond suture; the graft was removed using a closed graft stripper. After clearing muscle, fat, synovium, and whipstitching the other end, the diameters of the STG grafts were measured using the graft sizer available from the ConMed (Utica, New York)/Stryker (Kalamazoo, Michigan) graft sizer, which measured from a range of 3 mm to 12 mm with 0.5 mm increments. The minimum diameter that allowed the smooth passage of the entire graft through the tube completely was taken as the graft diameter. For the sake of consistency, any semitendinosus graft above 20 cm length was tripled as a rule unless a shorter length mandated it to be doubled. Gracilis was doubled in all cases irrespective of the length obtained. For ACL reconstruction, the same technique was used in all cases in which the femoral tunnel was drilled using a femoral jig at the footprint via the accessory anteromedial portal; the tibial tunnel was drilled using an appropriate tibial-zig at the footprint for anatomic single-bundle ACL reconstruction. The graft was fixed on the femur side using an Endobutton and the tibial side using an interference screw (titanium or bioabsorbable).

Following the surgery, a standard rehabilitation protocol was used for all patients, and patients were recalled for follow-up at six weeks, six months, one year, and two years. The patient’s knee range of motion, KOOS score, clinical tests, complications, and status of revision surgery were documented at each follow-up visit as part of data collection for the institutional knee registry. For patients who were not able to visit the hospital because of the COVID pandemic, a telephonic consultation was used to collect relevant data.

Data analysis

All characteristics were summarized descriptively. For continuous variables, the summary statistics of mean ± standard deviation (SD) were used. For categorical data, the number and percentage were used in the data summaries and diagrammatic presentation. The chi-square (χ2 ) test was used for the association between two categorical variables. The difference in the means of analysis variables between two independent groups was tested by an unpaired t-test. The difference in the means of analysis variables between more than two independent groups was tested by analysis of variance (ANOVA) and the F test of testing equality of variance. If the p-value was < 0.05, the results were considered to be statistically significant. Data were analyzed using SPSS software v.23 (IBM Corp., Armonk, NY) and Microsoft Office 2007 (Microsoft Corporation, Redmond, WA).

## Results

Out of the 144 patients enrolled in the study, 83.4% (120 of 144) of participants were males, and the remainder were females (16.6%; 24 of 144). The mean age of the patient was 30.89 ± 9.3 years (range 16-56). The graft size was divided into four ranges, that is, < 7, 7.1-8.0, 8.1-9, and > 9 mm. The mean age in each graft size range < 7, 7.1 -8, and 8.1-9 mm are 27.6 ± 10.3 years, 30.9 ± 9.9 years, 29.7± 7.8, and 30.5 ± 7.5 years, respectively. There was no statistically significant difference between the demographic variables between groups. The mean graft size in males and females was 8.23 ± 0.56 and 7.88 ± 0.54 (p=0.017), respectively. Most patients of both sexes had a graft between 7.1 and 8 mm. All the patients were followed up at least for one year to be included in the evaluation and the mean overall follow-up was 18.4 ± 4.5 months, and there was no difference in the mean follow-up period amongst patients with different graft diameters (p>0.05).

In our study, it was observed that patients with graft size < 7 mm reported an inferior postoperative functional outcome using the KOOS score at a follow-up period of one and two years in comparison to other graft sizes (p= 0.008 and p=0.007). No significant statistical differences were seen in the early postoperative phase in the outcome between the four groups of the graft sizes at the six weeks and six months follow-up. The functional outcome of patients with graft size 7.1-8.0, 8.1-9, and > 9 mm were similar, and there wasn’t much difference in the mean KOOS score, and the mean KOOS score for those > 7 mm was 88.3±8.5 (p<0.001) (Table 1).

**Table 1:** Mean KOOS among patients with graft diameters of < 7, 7.1-8.0, 8.1-9, and > 9 mm over preop, six weeks, six months, and one year Note: *significant at the 5% level of significance (p<0.05) KOOS: Knee Injury and Osteoarthritis Outcome Score

KOOS	Graft Size				Overall	p-value
	≤7	7.1-8.0	8.1-9.0	>9		
PREOP	59.8±9.6	58.5±7.5	58.2±7.3	60.2±6.3	58.5±7.6	0.797
6 WEEK	77.3±4.7	74.8±5.1	73.8±4.6	78±6.8	74.8±5	0.087
6 MONTH	79.2±6.8	83.1±6.8	83.4±6	85±5.4	82.8±6.6	0.110
1 YEAR	79.2±6.8	86.9±7.1	87.1±5.9	88.6±6.4	86.4±7.4	0.008*
2 YEAR	80.5±13.1	88.3±8.5	88.9±6.8	89.8±7.5	87.8±8.8	0.007*

On a detailed analysis of the KOOS scores at the two-year follow-up, patients with a graft diameter of ≤ 7mm did poorly, mainly with KOOS subscale scores of qualities of life and sports and recreation with a mean score of 74.3±9.5 and 61.7±7.8 (p=0.006 and p=0.013), respectively, whereas there was no statistically significant difference in the subscale score of pain and symptoms. The patients with graft diameter 7.1-8.0, 8.1-9, and > 9mm did well on all the KOOS subscale scores, and there were no differences between the means of subscale scores (Table 2). In our study, nine (6.2%) out of 144 patients underwent revision surgery of the ACL ligament. Among the revision cases for ACL-R, 55.6% (5) were in the age group of 21-30 years, followed by 22.2% (2), 11.1% (1) in 41-50 years, <20 years, and 31-40 years each, respectively. There was no positive statistical correlation seen with age group and incidence of revision ACL-R surgeries (p=0.742). Similarly, no significant association was seen between sex and incidence of ACL-R revision surgeries (p=0.644). However, we did observe a positive statistical co-relation between graft size and revision rates of ACL-R surgeries (p=0.027). It was observed the mean graft size of all cases that had undergone revision (7.78±0.67) was significantly less than those that did not require revision (8.40±0.56, p=0.014). Twenty-three point one percent (23.1%; 3 out 13) patients with graft < 7mm underwent revision, whereas only 5.8% and 2.6% of patients underwent revision with a graft diameter of 7.1-8.0 and 8.1-9.0 (p=0.027) (Table 3).

**Table 2:** The mean KOOS subscore, i.e., symptoms, pain activity of daily living, sports and recreation, and quality of life compared with patients with a graft diameter of < 7, 7.1-8.0, 8.1-9, and >9 mm at the two years follow-up Note: *significant at the 5% level of significance (p<0.05) KOOS: Knee Injury and Osteoarthritis Outcome Score

KOOS	Graft Size				p-value
	≤7	7.1-8.0	8.1-9.0	>9.0	
Symptoms	90.3±3.9	94.4±3.2	93.2±3.7	93.8±4.1	0.689
Pain	86.4±3.7	91.2±5.4	92.0±4.6	93.0±3.8	0.400
The activity of daily living	78.6±5.5	89.4±7.6	83.8±6.4	89.1±4.3	0.052
Sports and Recreation	61.7±7.8	84.3±6.9	85.4±6.1	83.8±8.5	0.013*
Quality of life	74.3±9.5	82.1±6.2	88.5±6.9	86.1±7.8	0.006*

**Table 3:** Revision rates compared for patients with a graft diameter of < 7, 7.1-8.0, 8.1-9, and > 9 mm at the two years follow-up Note: * significant at the 6% level of significance (p<0.05)

Graft Size	Total Cases	Revision Present		Revision Absent		p-value
		N	%	N	%	
≤7	13	3	23.1%	10	76.9%	0.027*
7.1-8.0	86	5	5.8%	88	94.2%	
8.1-9.0	34	1	2.6%	37	97.4%	
>9	11	0	0	11	100	
Total	144	9	6.3%	135	93.8%	
Mean Graft Size	8.17±0.57	7.78±0.67		8.40±0.56		0.014

## Discussion

One of the major drawbacks of a hamstring autograft is the variability in its size and the inability to accurately predict preoperatively. Though some authors have recommended a graft size of at least 7 mm, the ideal size of hamstring graft for ACL reconstruction has not been documented in the literature [22-24]. ACL reconstruction is commonly performed using hamstring graft for knee instability to improve the patient’s outcome, but the chances of graft failure are known to occur in around 1.8% to 10.8 % of the cases [25-26]. Although revision of ACL reconstruction is considered a marker of graft failure, many patients continue to have their daily activities affected without undergoing revision surgery. Thus, it is important to determine the improvement of functional outcomes to determine the success of ACL reconstruction surgery. Moreover, multiple contributors contribute to the failure of reconstruction like age, level of activity, surgical technique, etc., among which graft size is a major factor. The primary outcome of our study is to ascertain whether the size of the graft had any influence on the patient’s reported functional outcome and if the secondary outcome is the influence of this on revision surgery.

Magnussen et al., in one of the earlier studies, evaluated the effect of the size of the graft used in the revision as an indicator of graft failure. They concluded that hamstring autografts of size 8 mm in diameter or less are linked with an increase in the rate of revision. A major limitation conceded by them was that they did not consider the patient with graft failure and failed to undergo revision. Moreover, functional outcome was not determined in the study [21]. Park et al., in their study on 296 patients undergoing ACL reconstruction considered International Knee Documentation Committee (IKDC) grades C and D in addition to revision surgery as a marker of failure and found that patients with graft size < 8 mm had a 5.2% failure rate when compared to 0% in grafts ⩾ 8 mm. Among the 12 failures in their study, the majority of them had combined injuries and needed additional procedures along with ACL reconstruction, which may have contributed to the failure [22].

Mariscalco et al., in their retrospective study of 263 patients, considered the effect of graft size on KOOS. They concluded that smaller graft diameters were associated with poorer KOOS at the end of two years of follow-up. They noticed that there was a proportionate increase of 3.3-point in the KOOS pain scale, a 3-point increase in KOOS activity of daily living, and a 5.2-point increase in the KOOS sports function scale for every 1 mm increase in graft size [20]. In our study, we have tried to eliminate the confounding factors that were limiting factors in previous studies. We have excluded the patients with multiple ligament injuries, meniscal injuries, and chondral defects, which act as major confounding factors. Moreover, we have collected the functional outcome using KOOS throughout the follow-up period and compared the variation in the mean of functional outcome for different graft diameters. All the patients had similar improvements in functional outcomes in early follow-up at six weeks. But the patients with graft diameter ≤ 7mm had poorer functional outcomes at one year and two years when the patients tend to go back to a higher level of physical activity. On looking at the subscale of KOOS, patients with graft diameter ≤ 7mm had a mean sports and recreation subscale of 61.7±7.8 when compared to patients with >7 mm graft diameter who all had subscale score of > 83 (p=0.013*). The quality-of-life subscale value of KOOS was 74.3±9.5 for patients with graft diameter ≤7 mm, which was significantly less compared with those with graft diameter > 7 mm.

Most of the studies in the literature have considered the number of revision surgeries as the primary outcome measure to evaluate the effect of hamstring diameter among people undergoing ACL surgery. Park et al. observed no revisions in any of the patients with a graft diameter of 8 mm or more, whereas the revision risk was 5.2% among those with a graft diameter of less than 8 mm. Magnussen et al. had 14.3% (17 out of 119 patients) of cases undergoing revisions [21]. Among these, seven of 17, i.e., 41% of revisions had a graft diameter of ≤ 7 mm, and 82.4% (14 out of 17) had a graft diameter of ≤8 mm. But the major limitation of these studies is the small number of revisions. Even in our study, only nine out of 144 patients (6.3%) underwent revisions but the number of revisions was more in patients with a graft diameter of ≤7 mm, whereas only 5. % underwent revision among the patients with graft diameter between 8.1 and 9 mm. Considering the small number of revisions, it may be difficult to interpret and come to conclusions. Moreover, other factors determining the failure like level of the sporting activity, faulty technique, non-anatomical tunnel positions, etc. are not considered. We accept these limitations, which were also seen in the above studies. Thus, we have used revision rates only as a secondary outcome measure. Moreover, many patients may not undergo revisions despite poor functional outcomes and thus revision surgery of outcome measures may not give the true picture. There are many limitations to our study. The sizer used in our study was in increments of 0.5 mm, which decreased the accuracy of graft diameter measurement, but most centers, to our knowledge, use the same sizer to measure the graft diameter. As already discussed, a small number of revisions makes interpretation of results difficult. Moreover, there was a lack of information about the mechanism of failure, lack of quantitative measurement of laxity in the follow-up. Furthermore, the majority of our patients were males making the gender-based interpretation difficult. Thus, future studies should include multicentric studies with large samples ideally involving different races.

## Conclusions

The smaller hamstring graft diameter leads to poorer functional outcomes of patients’ ACL reconstruction, especially in sports and recreation. Though the number of revisions was high among those with a graft diameter of ≤7 mm, multicentric studies with a larger number of revisions are required to confirm the relation.
